# Superiority of Avacopan and Mepolizumab to Glucocorticoid Tapering in the Treatment of Anti-neutrophil Cytoplasmic Antibody (ANCA)-Associated Vasculitis: A Systematic Review

**DOI:** 10.7759/cureus.67161

**Published:** 2024-08-18

**Authors:** Rishma Gattu, Michelle Demory Beckler, Marc M Kesselman

**Affiliations:** 1 Osteopathic Medicine, Nova Southeastern University Dr. Kiran C. Patel College of Osteopathic Medicine, Fort Lauderdale, USA; 2 Microbiology and Immunology, Nova Southeastern University Dr. Kiran C. Patel College of Allopathic Medicine, Fort Lauderdale, USA; 3 Rheumatology, Nova Southeastern University Dr. Kiran C. Patel College of Osteopathic Medicine, Fort Lauderdale, USA

**Keywords:** monoclonal antibody, steroid, glucocorticoid, antineutrophil cytoplasmic antibody (anca) associated vasculitis (aav), mepolizumab, avacopan

## Abstract

Anti-neutrophil cytoplasmic antibody (ANCA)-associated vasculitis (AAV) comprises a spectrum of autoimmune diseases, including granulomatosis with polyangiitis (GPA), microscopic polyangiitis (MPA), and eosinophilic granulomatosis with polyangiitis (EGPA). Studies have shown that avacopan and mepolizumab are promising therapeutics for partial or complete replacement of glucocorticoids (GC), with sustained remission while completely weaning off GC. Avacopan inhibits C5aR in the complement pathway, preventing neutrophil migration, while mepolizumab targets IL-5R, reducing eosinophil activity. Additionally, complement inhibition has not only contributed to the recovery of renal function and alleviation of physical symptoms but has also enhanced patients' overall quality of life and mental well-being. This systematic review explores the pathogenesis of AAV, traditional treatments, and the potential of emerging complement and interleukin antagonist therapies such as avacopan and mepolizumab in revolutionizing AAV management.

## Introduction and background

Anti-neutrophil cytoplasmic antibody (ANCA)-associated vasculitis (AAV) is categorized into three types: granulomatosis with polyangiitis (GPA), previously known as Wegener’s granulomatosis, microscopic polyangiitis (MPA), and eosinophilic granulomatosis with polyangiitis (EGPA), previously known as Churg-Strauss syndrome. Among these vasculitides, MPA is the most common. Clinical manifestations of AAV occur in the respiratory and renal systems. Patients exhibit pauci-immune necrotizing crescentic glomerulonephritis, along with isolated sera showing either perinuclear or diffuse cytoplasmic staining patterns, corresponding to two types of autoantibodies: P-ANCA, targeting neutrophil myeloperoxidase (MPO), and C-ANCA, directed against neutrophil proteinase 3 (PR3). Typically, patients with the aforementioned clinical syndromes are positive for either P-ANCA or C-ANCA, but not both [1-3].

Epidemiology of AAV

Though MPA is the most prevalent form, AAV is a rare disease. In recent decades, several studies on its incidence and prevalence have been conducted, reporting a progressive worldwide increase over the past decade [4]. Globally, the annual AAV incidence ranges from 1.2 to 3.3 cases per 100,000 individuals, and the prevalence ranges from 4.6 to 42.1 cases per 100,000 individuals [4]. There is no clear gender predominance, though a slight male predominance among MPA compared to GPA has been reported [5,6]. Significant geographic differences have been reported among AAV subgroups, emphasizing how the incidence of GPA and EGPA increases with latitude [7]. MPO-AAV and MPA are more common in Japan. PR3-AAV and GPA are more common in Europe [4].

Complement system

Glucocorticoids (GC) are an adjunctive treatment for AAV, but they also act by systemically suppressing the underlying immune system. Despite effective results in some patients, even with treatment with GC, mortality rates remain higher than warranted due to infection, vasculitis activity, cardiovascular disease, and renal disease. A promising new target for AAV treatment is the complement pathway.

The complement system is a major component of both the innate and adaptive immune systems. Composed of three pathways, alternative, lectin, and classical, this system participates in a variety of immune functions, including pathogen lysis, pathogen opsonization, and inflammatory pathways. Each of the complement pathways functions in a signaling cascade-like fashion and is composed of whole proteins, enzymes, and zymogens. Dysregulation of complement is associated with a variety of pathologies, chief among these are autoimmune diseases.

Compared to other types of immune-mediated vasculitis, immune complex, and complement deposition are not readily visualized by light microscopy in AAV patients. While deposition can be visualized by electron microscopy, that deposition is not readily apparent suggests an alternative mechanism of disease initiation and progression. However, studies have shown an important role for complement in AAV other than its role in mediating immune complex-associated type III hypersensitivity reactions that underlie other types of vasculitis.

Inflammatory states and stimuli, like TNFa and prime neutrophils, lead to upregulated expression of MPO and PR3 by these cells. The binding of ANCA to these antigens can then lead to respiratory burst and degranulation, propagating inflammation. One of the first studies showing the role of complement in this inflammatory cascade found that inhibition of classical, alternative, and lectin complement pathways through knockout in mouse models of a common terminal complement component C5, resulted in protection from mouse-related human AAV-associated glomerulonephritis. Because all three complement pathways generate C5, studies then attempted to elucidate which complement pathway was involved in AAV [8].

Mouse models were initially used to specifically identify which complement pathway was involved. A role for the alternative pathway in AAV was found, while classical and lectin pathways were not found to be involved. This is supported by human studies showing higher levels of the alternative pathway components Bb and properdin in AAV patients compared to control. The levels of Bb and properdin correlate with an increased Birmingham Vasculitis Activity Score (BVAS) [9]. Additionally, levels of the alternative pathway regulator factor H have been shown to inversely correlate with AAV activity in patients, and dysregulated factor H activity has been reported. Combined, these data suggest that loss of complement inhibition may allow dysregulated alternative pathway activation and generation of pro-inflammatory factors, such as the anaphylatoxin C5a.

Anaphylatoxins, including C5a and C3a, are potent chemotactic factors that modulate the migration of neutrophils, macrophages, and monocytes as well as increase adhesion molecule expression on these cells. Thus, these molecules serve as important mediators of inflammatory responses by inducing and recruiting immune cell migration to sites of complement activation. As such, C5a has been implicated in AAV. In vitro, C5a can prime neutrophils, similar to TNFa, leading to upregulation of ANCA antigen expression. Additionally, in mouse models, wild-type bone marrow transplantation showed induction of necrotizing crescentic glomerulonephritis (NCGN), a murine model of AAV, whereas C5a receptor-deficient bone marrow transplantation abrogated this effect, suggesting an important role for both C5a and C5a receptor in AAV onset [10]. Indeed, oral administration of a C5a receptor antagonist to mice expressing human C5a receptor led to abrogation of NCGN in a dose-dependent manner [11]. These results were some of the first that strongly suggested C5a and C5a receptors as ideal targets for AAV treatment. Indeed, a newer monoclonal antibody therapy, avacopan, that targets the C5a receptor is now recommended for AAV.

While avacopan treats MPA and GPA, interestingly, EGPA works via a different mechanism. Another monoclonal therapy, mepolizumab, has been created to target a cytokine involved in eosinopoiesis. Both drugs involve a complex pathogenesis.

Pathogenesis

Th17 lymphocytes activate cytokines to prime neutrophils. C5a made through the alternative pathway stimulates neutrophils to display ANCA antigens that circulating ANCAs bind. The Fc (fragment crystallizable) region of ANCAs binds to receptors on neutrophils, causing overactivation. Overactivated neutrophils generate neutrophil extracellular traps (NETs), leading to inflammation and tissue damage. MPO and PR3 trigger more ANCA production through T helper cells, worsening vasculitis.

In MPA and GPA, ANCA activates neutrophils to attack vessels via granules and NETs. ANCA-mediated inflammation includes C5a and inflammatory cytokines priming neutrophils, which activate B cells to produce ANCA. The ANCA attaches to the neutrophils by Fc gamma or MPO, and then this process leads to ROS and NET production.

Avacopan, previously known as CCX168, functions as an allosteric antagonist of the C5a receptor (C5aR) in the alternative pathway of the complement system. Through its inhibition of the C5a/C5aR axis, avacopan is anticipated to have minimal impact on the formation of the membrane attack complex, which includes C5b. Consequently, this is expected to result in little influence on the innate immune response in patients undergoing treatment with avacopan [12].

However, the role of ANCA in EGPA is unknown. Rather, IL-5 is involved in the pathogenesis of EGPA. Th2 produces IL-5, causing differentiation of eosinophils and inhibiting eosinophilic apoptosis. Activation of eosinophils causes the release of cytotoxic granules, inducing inflammation and tissue damage. In clinical manifestations, patients had a history of bronchial asthma and eosinophilia, but low creatinine, which is unusual compared to other AAVs [13]. Mepolizumab is an IgG1 antibody that binds IL-5 to prevent binding to its receptor on eosinophils, reducing blood eosinophil levels under 500 eosinophils/microliter [14]. These therapies focus on reducing GC dependence.

Traditional treatments and their drawbacks

Steroids, like GC, reduce inflammation and suppress the immune system, with therapeutic effects that are valuable in autoimmune diseases. Chronic immune suppression, however, leads to vulnerability to infection and other side effects [15]. The search for treatment alternatives to GC has led to the addition of immunosuppressive medications in the treatment regimen: disease-modifying anti-rheumatic drugs (DMARDs) including azathioprine (AZA), methotrexate, and mycophenolate mofetil (MMF) disrupt DNA or nucleotide synthesis, and impact dividing cells, including pathogenic lymphocytes.

Prior to 2011 the standard treatment for ANCA-related vasculitis involved initially using the immunosuppressant cyclophosphamide (CYC) for three to six months, followed by AZA for one year or more [16]. Due to the toxicity of CYC, rituximab (RTX) became an alternative for induction treatment after receiving approval from the FDA in 2011 [17,18].

ANCAs play a role in AAV by stimulating neutrophils to release B cell activating factor, enhancing the survival of autoreactive B cells. RTX is a monoclonal antibody targeting CD20R on B cells, leading to the depletion of B cells expressing this surface marker. This process prevents B lymphocytes from releasing ANCA and from activating T lymphocytes that prime neutrophils to display ANCA antigens [8]. Due to this activity, RTX is now preferred over AZA for maintenance therapy. In the MAINRITSAN (Maintenance of Remission Using Rituximab in Systemic ANCA-Associated Vasculitis) 1 and RITAZAREM (Rituximab Versus Azathioprine After Induction of Remission With Rituximab for Patients With ANCA-Associated Vasculitis) trials, RTX achieved longer remission periods than AZA and in the MAINRITSAN trial 2, patients exhibited lower relapses with RTX maintenance therapy over standard therapy [18-20]. However, two trials have shown that RTX is not inferior to CYC in inducing remission in both new and relapsed patients with GPA and MPA [21,22].

While this regimen often effectively treats vasculitis, relapses are frequent, and the need for GC persists. Chronic GC use introduces an array of adverse effects, including increased cardiovascular mortality, Cushing syndrome, aseptic joint necrosis, systemic immunosuppression, and cataracts. Floyd et al. utilized a universal quantifiable tool to assess GC toxicity [15]. In a population with an average age of 65.9 years, GC outcomes and levels were measured when concomitantly treated with CYC or RTX for AAV patients. There were no statistical differences observed in GC dose and toxicity in patients receiving RTX vs CYC. However, during the four-year follow-up, patients encountered adverse effects: 43.9% infection, 24.4% reduced bone density, 24.4% increase in weight over 5 kilograms, 17.1% diabetes mellitus, 17.1% blood pressure increase over 20 mmHG, 12.2% gastrointestinal side effects, 7.3% increase in statin dose, 7.3% fragility fracture, 2.4% skin changes. Patients with a GTI (Glucocorticoid Toxicity Index) score over 60 had received over 9000 mg of GC. This study strongly suggests a positive correlation between steroid dose and side effects [15].

Methods

**Figure 1:**
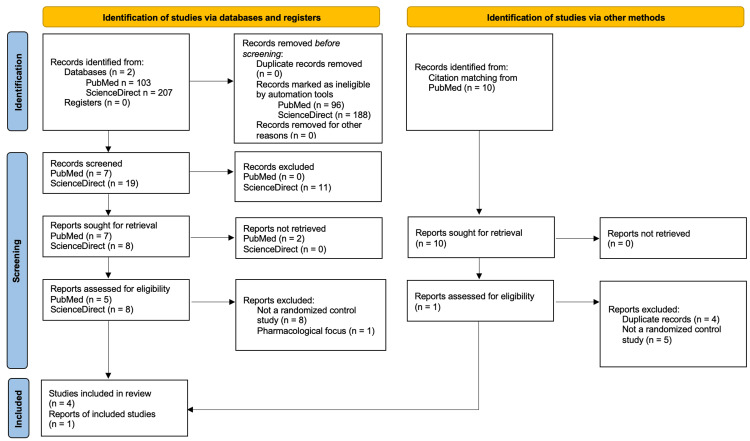
PRISMA flow diagram detailing the study identification and selection for avacopan in the treatment of AAV. PRISMA: Preferred Reporting Items for Systematic Reviews and Meta-Analyses, AAV: antineutrophil cytoplasmic antibody-associated vasculitis.

**Figure 2:**
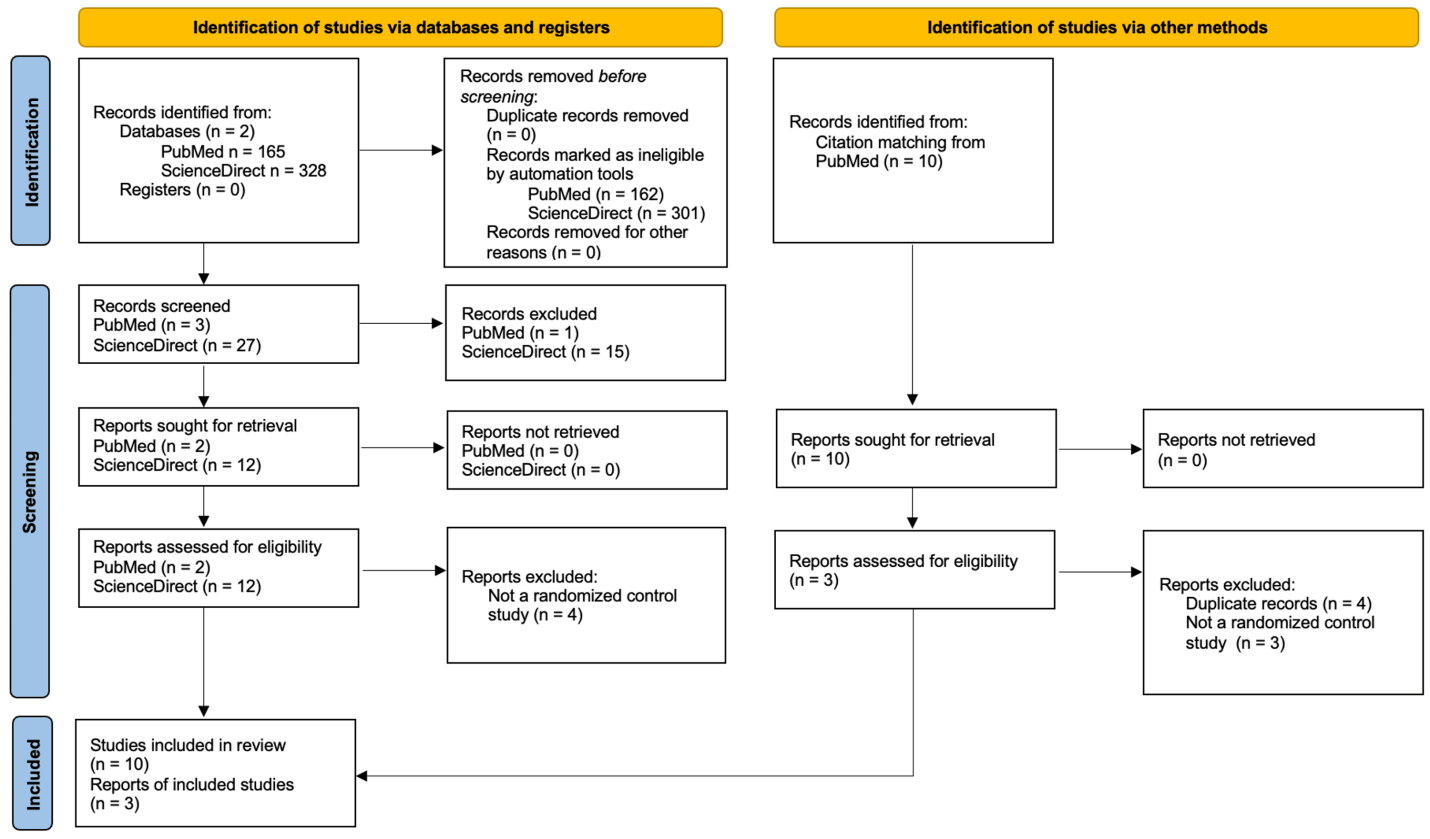
PRISMA flow diagram detailing the study identification and selection for mepolizumab in the treatment of AAV. PRISMA: Preferred Reporting Items for Systematic Reviews and Meta-Analyses, AAV: antineutrophil cytoplasmic antibody-associated vasculitis.

A systematic review was performed to compare avacopan or mepolizumab to GC. A comprehensive literature search was conducted in electronic databases, including PubMed and Elsevier from inception to January 2024. The search terms included “Avacopan in the treatment of ANCA-associated vasculitis” and “Mepolizumab in the treatment of ANCA-associated vasculitis”. Filters were applied to limit the search to human studies published in English. Studies were considered eligible if they met the following criteria: randomized control, retrospective study (only for mepolizumab), and free full text. Titles and abstracts were screened for potential eligibility, followed by a full-text review of selected articles. Additional articles were found through citation matches on PubMed. The study selection process is visually represented in the PRISMA (Preferred Reporting Items for Systematic Reviews and Meta-Analyses) flow diagrams for avacopan (Figure 1) and mepolizumab (Figure 2), outlining the number of records identified, screened, assessed for eligibility, and included in the final analysis.

## Review

Multiple studies have demonstrated that immunoglobulin therapy can replace GC in standard of care (SoC) treatment to some extent. Even in the few articles with no statistical significance, there appeared to be a difference in adverse events that favor avacopan and mepolizumab. Relapse rates were a key indicator in determining medication success. Specifically, the Birmingham Vasculitis Activity Score (BVAS) is a tool to quantify the severity of disease for various types of vasculitis. Comprising scored components categorized into nine organ systems, the BVAS encompasses a wide range of clinical manifestations associated with vasculitis [23]. The following studies will use this tool to measure clinical outcomes.

Avacopan efficacy

Based on remission rates, which denote a BVAS of 0, Jayne et al. indicated that avacopan demonstrates noninferiority to prednisone in achieving remission at week 26, but superiority in sustaining remission at week 52 [24]. A total of 16 of 158 patients (10.1%) in the avacopan group and 33 of 157 patients (21.0%) in the prednisone group had relapses. This is a pivotal finding, as the ability to reduce or replace high-dose GC is highly desired. Additionally, Jayne et al. showed that avacopan treatment is associated with improved renal inflammation and reduced adverse effects, such as diabetes, psychiatric disorders, and weight gain [25]. This finding is promising, as it not only suggests that avacopan could improve patient outcomes but also indicates a means of mitigating the adverse effects of traditional treatments and supports the idea that avacopan can effectively replace high-dose GC.

Renal recovery with avacopan

Cortazar et al. indicate that avacopan had a positive impact on renal recovery in a subgroup of patients with baseline glomerular filtration rate (GFR) ≤20 ml/min per 1.73 m2 [26]. Avacopan was also associated with a notable improvement in estimated GFR (eGFR) and a potentially lower rate of serious adverse events compared to prednisone in this specific subgroup [26]. This result signifies the potential of avacopan in improving renal function in a vulnerable patient subset.

In many studies, avacopan groups led to improvement in GFR, emphasizing its potential to reduce renal involvement in AAV [24-28]. The results suggest that avacopan can play a significant role in reducing the dependence on GC, particularly in cases where renal function is a concern.

In both avacopan groups of Jayne et al., albuminuria decreased, and urinary creatinine-corrected monocyte chemoattractant protein-1 (MCP-1) levels improved significantly when compared to the control group at weeks 4 and 12 of the CLEAR (A Study to Evaluate the Safety and Efficacy of CCX168 in Subjects With ANCA-Associated Vasculitis) trial [25]. Notably, patients in the avacopan groups experienced a lower incidence of adverse effects (34%) compared to the control group (65%).

Tesar and Hruskova noted that in the CLEAR trial, patients with AAV who were treated with avacopan without corticosteroids experienced an enhanced quality of life (QoL), improvements in vitality (reduced fatigue), and a lower incidence of adverse events [27]. Specifically, they had fewer side effects associated with corticosteroid use, such as psychiatric disorders, new onset or worsening diabetes, weight gain, fractures, and cataracts. This indicates that avacopan could be an effective steroid-sparing alternative for these patients [27].

Merkel et al. showed an improvement in eGFR (+6.2 ml/min/1.73m2 for avacopan 30 mg, +1.3 ml/min/1.73m2 for avacopan 10 mg, and +2.0 ml/min/1.73m2 for SoC only), renal response (63% for avacopan 30 mg, 40% for avacopan 10 mg, and 17% for SoC only) [28]. The avacopan 30 mg group demonstrated the highest rates of early disease remission, renal responses in patients with hematuria and albuminuria, and improvements in renal function as measured by eGFR. Additionally, avacopan 30 mg exhibited the most substantial improvements in eGFR, MCP-1, and CRP (C-reactive protein) levels, suggesting its effectiveness in addressing these aspects of the condition.

Geetha et al. showed renal recovery as well [29]. At the 52-week mark, 71% of individuals in the avacopan cohort and 56.1% in the prednisone tapering group achieved sustained remission. These findings indicate that among patients with ANCA-associated vasculitis undergoing RTX-based induction therapy, avacopan exhibits a favorable safety profile and is linked to enhanced restoration of renal function, quicker reduction in albuminuria, and reduced glucocorticoid-related side effects when compared to a prednisone taper.

Avacopan's impact on QoL

Merkel et al. highlight the potential of avacopan to enhance health-related quality of life (HRQoL) in patients with AAV [28]. The inclusion of avacopan alongside SoC resulted in notable enhancements in different dimensions of HRQoL, including reductions in bodily pain, increased vitality, better mental health, improved physical functioning, and enhanced emotional well-being. Moreover, the 30 mg dose exhibits the most substantial improvements in disease markers, including renal function. While the impact on disease remission and renal function varied, these data suggest that avacopan can be a safe addition to the AAV treatment arsenal. This result raises the possibility of integrating avacopan with existing treatment regimens to enhance patient outcomes.

Tavneos® (avacopan) has been FDA-approved as a 10 mg capsule for the treatment of GPA and MPA [12]. This drug was approved based on data from the ADVOCATE (A Phase 3 Clinical Trial of CCX168 (Avacopan) in Patients With ANCA-Associated Vasculitis) trial [26]. When compared to standard therapy Tavneos® decreased GC use by 81% and showed an improvement in mental health at weeks 26 and 52. The findings on avacopan are summarized in Table 1.

**Table 1:** Summary of studies using avacopan in the treatment of AAV. mg = milligrams, BID = twice daily, SoC = standard of care, eGFR = estimated glomerular filtration rate, BVAS = Birmingham Vasculitis Activity Score, VDI = Vasculitis Damage Index, HRQoL = Health-related quality of life, AAV = anti-neutrophil cytoplasmic antibody (ANCA)-associated vasculitis.

Study title	Author	Methods	Results	Conclusion
Avacopan for the treatment of ANCA-associated vasculitis	Jayne DR et al. (2021) [24]	166 patients received 30 mg avacopan BID and 165 received 60 mg prednisone tapered to 0 mg. Both groups also received cyclophosphamide followed by azathioprine or rituximab.	At week 26, remission was achieved by 72.3% in the avacopan group and 70.1% in the prednisone group. At week 52, sustained remission was 65.7% for avacopan and 54.9% for prednisone.	Avacopan was not inferior to prednisone at week 26 and was superior at week 52 for sustained remission.
Randomized trial of C5a receptor inhibitor avacopan in ANCA-associated vasculitis	Jayne DR et al. (2017) [25]	The CLEAR trial involved 67 patients receiving either a placebo plus 60 mg prednisone taper, 30 mg avacopan BID plus 20 mg prednisone, or 30 mg avacopan BID without prednisone. All received cyclophosphamide or rituximab.	At week 12, clinical response was achieved by 70% of control patients, 86.4% of the avacopan plus reduced-dose prednisone group, and 81% of the avacopan without prednisone group. Remission at week 4 sustained to week 12 occurred in 21% of the avacopan group versus 5% of the control group.	Avacopan can effectively and safely replace high-dose glucocorticoids, showing faster renal inflammation improvement without immediate rebound effects.
Renal recovery for patients with ANCA-associated vasculitis and low EGFR in the advocate trial of avacopan	Cortazar FB et al. (2023) [26]	Patients from the ADVOCATE trial with a baseline eGFR ≤20 ml/min/1.73 m².	At week 52, eGFR increased by 16.1 ml/min/1.73 m² in the avacopan group versus 7.7 ml/min/1.73 m² in the prednisone group. Increases were more than double for 41% of avacopan patients and 13% of prednisone patients.	Avacopan resulted in greater improvement in eGFR compared to prednisone for patients with baseline eGFR ≤20 ml/min per 1.73 m2.
Adjunctive treatment with avacopan, an oral C5a receptor inhibitor, in patients with antineutrophil cytoplasmic antibody-associated vasculitis	Merkel PA et al. (2020) [28]	A 12-week study with avacopan (10 mg or 30 mg) plus SoC versus SoC alone.	High BVAS response across all groups, with lower VDI for avacopan plus SoC compared to SoC alone. Early remission, eGFR, renal recovery, and HRQoL was higher in the avacopan 30 mg group.	Avacopan, especially at 30 mg, improved remission time and was well tolerated.
Efficacy and safety of avacopan in patients with ANCA-associated vasculitis receiving rituximab in a randomised trial	Geetha D et al. (2023) [29]	Avacopan 30 mg BID or 60 mg tapered prednisone over 52 weeks with background rituximab therapy.	Remission at week 26 was 77.6% for avacopan and 75.7% for prednisone. Sustained remission at week 52 was 71% for avacopan versus 56.1% for prednisone.	Avacopan demonstrated better outcomes in relapse rates, renal function recovery, and reduced glucocorticoid toxicity compared to prednisone in patients with rituximab induction therapy.

Mepolizumab efficacy

Mepolizumab, an IL-5 inhibitor, is used primarily for treating severe eosinophilic asthma and EGPA. This drug, as discussed in the MIRRA (A Study to Investigate Mepolizumab in the Treatment of Eosinophilic Granulomatosis With Polyangiitis) trial in Wechsler et al., extended remission periods and reduced the annualized relapse rate of AAV patients [30]. The data revealed that mepolizumab treatment allowed for lower prednisolone or prednisone doses, thus reducing the reliance on GC. Specifically, 28% of participants in the mepolizumab group achieved 24 or more weeks of remission, whereas only 3% of those in the placebo group reached this level. Furthermore, a higher percentage of participants in the mepolizumab group remained in remission at both week 36 and week 48 (32% vs. 3% in the placebo group). Remission was not achieved in 47% of the participants receiving mepolizumab, compared to 81% in the placebo group. The annualized relapse rate was significantly lower in the mepolizumab group (1.14) compared to the placebo group (2.27). Furthermore, mepolizumab led to improved QoL and reduced corticosteroid-related side effects as 44% of participants in the mepolizumab group maintained a daily prednisolone or prednisone dose of 4.0 mg or less during weeks 48 through 52, whereas only 7% in the placebo group achieved this low dose. While the focus of this study was on reducing asthma exacerbations, the implications for reducing corticosteroid dependence are evident. This finding is particularly relevant for patients with AAV who experience concomitant asthma.

While there was no statistically significant difference in the occurrence of adverse events between the mepolizumab group and the placebo group, with 97% and 94% of participants experiencing adverse events, respectively, it is important to note that there was an imbalance in the occurrence of serious adverse events. Specifically, the steroid group had a higher incidence of serious adverse events, which included significant asthma-related events.

In a retrospective study performed by Ríos-Garcés et al., the longest follow-up period to date was observed among any reported cohort with a mean time of 38 months [31]. All individuals attained a BVAS of 0 points within 12 months. Patients had fewer flares, which were related to asthma or ENT manifestations. Disease activity improvement led to significant GC reduction. Mepolizumab resulted in an annualized relapse rate of 0.51, with asthmatic flares predominating and no vasculitic flares. In comparison, the MIRRA trial reported a higher rate of 1.14 [30].

Vergles et al. and Vultaggio et al. show increased remission and decreased asthma exacerbations [32,33]. Though Vergles et al. was a case study, it revealed that patients experienced reduced peripheral blood eosinophil counts, better asthma control, and a reduction in relapse rates [32]. In Vultaggio et al., 66.6% experienced no asthma exacerbations [33]. After 12 months of treatment, clinical remission (BVAS of 0) was achieved by a substantial majority of patients, specifically 17 out of 18 patients [33]. Rhinosinusitis improved, and there were reductions in eosinophil-derived neurotoxin (EDN) and eosinophil cationic protein (ECP) levels. Additionally, half of the patients displayed improved forced expiratory volume in one second (FEV1). A majority of patients (77.7%) were able to reduce their daily oral corticosteroid (OCS) dose by at least 50%. By month 12, most patients (77.7%) had successfully reduced their OCS dose to an average of 3.8 ± 0.6 mg of prednisone per day, with three patients (16.6%) completely discontinuing OCS therapy. These findings highlight the positive impact of mepolizumab in reducing asthma exacerbations and the need for oral corticosteroids in this patient population.

In Kim et al. patients treated with mepolizumab achieved successful tapering of corticosteroids and decreased eosinophils [34]. There was a decrease in the mean steroid dose from 12.9 to 4.6 mg/d after 12 weeks of therapy. Although clinical stability was maintained and overall prednisone dosing decreased, there were no significant changes in markers of disease activity, and mean IL-5 levels remained unchanged in response to mepolizumab therapy. Despite a relatively small sample size and lack of assessment of airway eosinophilia, this study suggests that adjuvant therapy with mepolizumab may enable substantial corticosteroid tapering while keeping patients clinically stable. Notably, there was a decrease in the mean steroid dose from 12.9 mg/day to 4.6 mg/day after 12 weeks of mepolizumab therapy, indicating the potential benefits of this approach in reducing steroid dependence.

Steinfeld et al. conducted a post hoc analysis that acknowledged the clinical benefits of mepolizumab [35]. In this study, endpoints were delineated to be at least a 50% reduction in GC dose in weeks 48-52, lack of relapses, and remission during any point. Remission was defined to be BVAS=0 and GC dose ≤4 mg/day (clinical benefit 1) or ≤7.5 mg/day (clinical benefit 2). Patients treated with mepolizumab displayed significant clinical benefits compared to those who received a placebo (78% vs 32% for clinical benefit 1 and 87% vs 53% for clinical benefit 2). Additionally, 29% of patients in the mepolizumab group met all three endpoints, whereas only 7% of the placebo group achieved this level of response, highlighting the efficacy of mepolizumab in achieving remission and decreasing GC dependence.

Mepolizumab's impact on QoL

In the retrospective study by Özdel et al., patients were treated with mepolizumab at a dosage of 100 mg per month, which led to a substantial decrease in the daily dose of oral corticosteroids, dropping from 11.04 mg to 3.65 mg [36]. Patients reported improvements measured by multiple QoL questionnaires. The mean forced expiratory volume in 1 second (FEV1) increased from 1.88 L at baseline to 2.46 L at the 12th month, indicating improved lung function. A large percentage of patients responded well to treatment, with 76% achieving a complete response at the 6th month and 81.25% at the 12th month.

Long-term follow-up and ongoing studies

Wang et al. included a 12-month 100 mg MEP quadri-weekly subcutaneous injection that also led to improved outcomes, with six patients forgoing steroids completely after a certain period [37]. Similarly, in Ramirez et al., five patients were able to forgo GC [38].

In an ongoing 96-week study, patients with EGPA who had received four-weekly subcutaneous mepolizumab 300 mg for ≥96 weeks before entering the study exhibited a reduction in GC dose from 6.9 (pre-mepolizumab) to 3.0 (baseline) and 2.0 mg/day (weeks 45-48) [39]. The percentage of patients not taking oral steroids increased from 8% to 32% and 38%, respectively. The prevalence of patients experiencing clinical symptoms decreased from 94% (pre-mepolizumab) to 73% (baseline) and 67% (week 48).

On November 4, 2015, Nucala® (mepolizumab) by GlaxoSmithKline became the first IL-5 antagonist approved by the FDA for add-on maintenance treatment for eosinophilic asthma in individuals aged 12 years and older [14]. Three trials evaluated mepolizumab for asthma: DREAM (Dose Ranging Efficacy and Safety With Mepolizumab in Severe Asthma), MENSA (Efficacy and Safety Study of Mepolizumab Adjunctive Therapy in Subjects With Severe Uncontrolled Refractory Asthma), and SIRIUS (Mepolizumab Steroid-Sparing Study in Subjects With Severe Refractory Asthma) (the latter two were confirmatory studies). Mepolizumab was administered every four weeks as an additional treatment alongside the ongoing background asthma therapy. The data illustrated that mepolizumab significantly reduces exacerbations, including those requiring hospitalization or emergency visits, and is more than twice as likely to reduce glucocorticoid doses compared to placebo [40,41,42]. In 2017, Nucala® gained approval for EGPA treatment following the MIRRA trial [14]. The findings on mepolizumab are summarized in Table 2.

**Table 2:** Summary of studies using mepolizumab in the treatment of EGPA. mg = milligrams, IV = intravenous, SC = subcutaneous, EGPA = eosinophilic granulomatosis with polyangiitis, FEV1 = forced expiratory volume in one second, BVAS = Birmingham Vasculitis Activity Score, OCS = daily oral corticosteroid.

Study title	Author	Methods	Results	Conclusion
Mepolizumab or placebo for eosinophilic granulomatosis with polyangiitis	Wechsler ME et al. (2017) [30]	68 participants received 300mg mepolizumab and 68 placebo monthly for 52 weeks. All had stable prednisolone/prednisone doses.	28% of the mepolizumab group and 3% of the placebo group had greater than 24 weeks of remission. Mepolizumab led to more weeks of remission and higher remission rates at weeks 36 and 48 compared to placebo. Additionally, 44% of the mepolizumab group and 7% of the placebo group were able to reduce their daily prednisolone dose to 4.0 mg or less by 52 weeks.	Mepolizumab increased remission weeks and reduced glucocorticoid use.
Response to mepolizumab according to disease manifestations in patients with eosinophilic granulomatosis with polyangiitis.	Ríos-Garcés R et al. (2022) [31]	11 patients were treated with varied doses of mepolizumab and remission induction with prednisone at a mean dose of 11.36mg/day.	All patients achieved BVAS 0 at 12 months, with fewer and milder flares, improved asthma/ENT manifestations, and notable glucocorticoid tapering.	Mepolizumab allowed significant glucocorticoid reduction and maintained disease control.
Mepolizumab as a glucocorticoid-sparing agent in eosinophilic granulomatosis with polyangiitis (EGPA): is a lower dose sufficient?	Vergles M et al. (2021) [32]	100 mg of mepolizumab monthly, with tapering glucocorticoids and discontinuing immunosuppressants as tolerated.	Decreased BVAS, increased asthma control scores, and sustained relapse-free periods with low doses of mepolizumab.	Low-dose mepolizumab effectively allowed glucocorticoid tapering and improved asthma control.
Low-dose mepolizumab effectiveness in patients suffering from eosinophilic granulomatosis with polyangiitis	Vultaggio A et al. (2020) [33]	100 mg/4 weeks mepolizumab was administered for 12 months in 18 patients with severe asthma. Symptoms, exacerbation rates, lung function, and remission rates were monitored.	Clinical remission was achieved in 94.4% of patients, with reduced eosinophil counts, lower asthma exacerbations, and reduced steroid doses by half.	Low-dose mepolizumab was effective in improving asthma outcomes and reducing steroid use.
Mepolizumab as a steroid-sparing treatment option in patients with Churg-Strauss syndrome	Kim S et al. (2010) [34]	7 patients received 4 monthly doses of 750 mg mepolizumab IV.	Mean steroid dose decreased from a mean of 12.9 to 4.6 mg/d, with reduced eosinophil counts and stable clinical markers by week 12.	Mepolizumab allowed significant steroid tapering without increasing disease activity.
Evaluation of clinical benefit from treatment with mepolizumab for patients with eosinophilic granulomatosis with polyangiitis	Steinfeld J et al. (2019) [35]	Patients received 300 mg mepolizumab or placebo every 4 weeks for 52 weeks with a stable steroid dose of 7.5 to 50 mg/d.	78-87% of mepolizumab patients experienced higher remission rates, lower relapse rates, and a 50% reduction in steroid doses over the placebo group.	Mepolizumab allowed for successful steroid tapering and increased remission rates.
Effectiveness of low-dose mepolizumab in the treatment of eosinophilic granulomatosis with polyangiitis (EGPA): a real-life experience	Özdel Öztürk B et al. (2022) [36]	Mepolizumab 100 mg/month, with dose increase to 300 mg/month in some cases.	Reductions in OCS doses from 11.04 to 3.65 mg/d, improved asthma and sinonasal outcomes, and reduced blood eosinophil counts. At month 6 and 12, 76% and 81.25%, respectively, responded completely.	Low-dose mepolizumab was effective in improving patient outcomes and reducing steroid use.
Monocentric study of IL-5 monoclonal antibody induction therapy for eosinophilic granulomatosis with polyangiitis	Wang CR et al. (2024) [37]	10 patients received a 12-month induction therapy with 100 mg quadri-weekly injections.	Reduced BVAS with complete or partial remission in all patients.	Induction therapy with mepolizumab was effective and safe for active and relapsing EGPA patients.
Real-life efficacy and safety of mepolizumab for eosinophilic granulomatosis with polyangiitis	Ramirez GA et al. (2022) [38]	Monthly 300 mg mepolizumab, with prednisone tapering with a median dose of 8.75 mg/d.	Significant decrease in BVAS, with most patients reducing prednisone dose or discontinuing it. Improved asthma symptoms and fewer eosinophil-dependent manifestations. All but one patient achieved remission by last follow-up. All patients were able to taper prednisone to a mean dose of 2.5mg/d with 5/14 completely off steroid by the last visit.	Mepolizumab was effective in reducing prednisone use and improving disease control.
Real-world safety and effectiveness of mepolizumab for patients with eosinophilic granulomatosis with polyangiitis in Japan: a 48-week interim analysis of the MARS study	Ishii T et al. (2023) [39]	Ongoing 96-week study, patients received 300 mg SC mepolizumab every 4 weeks.	The median steroid dose decreased from 6.9 to 3.0 mg/day and further to 2.0 mg/day during Weeks 45-48. The proportion of patients not using steroids increased from 8% to 32% and then to 38% during the same periods. The percentage of patients with clinical symptoms decreased from 94% to 73% and then to 67% by Week 48.	The results after 144 weeks of mepolizumab treatment confirmed the drug’s known safety profile, enabled significant reductions in steroid doses, and improved disease control.
Mepolizumab for severe eosinophilic asthma (DREAM): a multicentre, double-blind, placebo-controlled trial	Pavord I et al. (2012) [40]	one of three doses of IV mepolizumab delivered monthly (75 mg, 250 mg, or 750 mg) or matched placebo (100 mL of 0.9% NaCl); 159 participants in the placebo group, 154 in the 75 mg mepolizumab group, 152 in the 250 mg group, and 156 in the 750 mg group.	The annual rate of clinically significant exacerbations was 2.40 per patient in the placebo group. For the 75 mg mepolizumab group, the rate was 1.24, representing a 48% reduction. The 250 mg group had a rate of 1.46, showing a 39% reduction. The 750 mg group had a rate of 1.15, indicating a 52% reduction.	Mepolizumab is effective and well-tolerated in reduction of asthma exacerbations.
Mepolizumab treatment in patients with severe eosinophilic asthma (MENSA trial)	Ortega HG et al. (2014) [41]	Confirmatory trial- mepolizumab was given monthly for 32 weeks to 576 patients; Participants received either SC mepolizumab 100 mg, IV mepolizumab 75 mg, or a placebo, along with their regular asthma medications.	Patients treated with either subcutaneous or intravenous mepolizumab had a longer duration before their first asthma exacerbation compared to those on placebo. The rate of asthma exacerbations was reduced by 47% with IV mepolizumab and by 53% with SC mepolizumab compared to placebo. The need for emergency department visits or hospitalizations due to exacerbations decreased by 32% in the intravenous group and by 61% in the subcutaneous group. At 32 weeks, the increase in FEV1 was 100 ml greater in the IV mepolizumab group and 98 ml greater in the SC mepolizumab group compared to placebo.	IV or SC mepolizumab effectively reduced asthma exacerbations and improved overall asthma control markers.
Oral glucocorticoid-sparing effect of mepolizumab in eosinophilic asthma (SIRIUS trial)	Bel EH et al. (2014) [42]	Confirmatory trial- 135 patients given mepolizumab 100 mg SQ or placebo monthly for 20 weeks. Steroids were decreased by 1.25 to 10 mg per day every 4 weeks. All participants continued their background asthma medications throughout the study duration.	The glucocorticoid dose was reduced by half in the mepolizumab group compared to no reduction in the placebo group. Patients receiving mepolizumab were 2.39 times more likely to reduce their glucocorticoid dose compared to those on placebo. Mepolizumab also led to a 50% median reduction in glucocorticoid dose from baseline, whereas no reduction was observed with placebo. The rate of exacerbations reduced by 32% in mepolizumab-treated patients compared to placebo.	Mepolizumab demonstrated a reduction in the need for daily oral glucocorticoid therapy in asthma patients, along with fewer exacerbations and improved control of asthma symptoms.

Future studies

While traditional therapies have demonstrated efficacy, they often come with increased morbidity. By targeting specific receptors rather than broad immunosuppression, complement inhibition offers a promising avenue with a better safety profile.

Several complement inhibitors have shown promise in AAV cases, prompting further examination: In a case study, non-selective inhibition of complement using an anti-C5 antibody, eculizumab, resulted in enhanced renal function in a severe AAV patient when combined with rituximab in a corticosteroid-free treatment regimen [43]. Similar to avacopan, vilobelimab is an antibody targeting C5a that presents a potential area for exploration [27]. When managing refractory asthma or ENT manifestations in EGPA, benralizumab seems to be a viable therapeutic option [44]. These new medication approaches underscore the evolving landscape of treatment options.

Additional experiments should investigate the impact of other monoclonal antibodies involved in the pathogenesis of AAV. Of the few randomized control studies on AAV, many lacked a long time period and a large cohort group. Moreover, exploring interventions in patients with low GFRs below 15 mL/min/1.73 m2 may advance our understanding and management of these conditions since many studies lack evidence on severely diseased cohorts. Due to the meager literature in this field of interest, future studies should prioritize using monoclonal antibodies over longer periods of time, in larger patient cohorts, and across varying disease severities.

## Conclusions

The emerging therapies avacopan and mepolizumab show promise in anti-neutrophil cytoplasmic antibody (ANCA)-associated vasculitis (AAV) management by reducing the use of glucocorticoids (GC) and improving patient outcomes. The data shown in the reviewed articles collectively suggest that these therapies have the potential to revolutionize AAV treatment. Nevertheless, it is essential to consider the limitations, such as varying sample sizes and conflicting statistical significance in some studies. Further research, including larger-scale studies, is warranted to confirm the long-term efficacy and safety of these therapies. This discussion contributes to the evolving landscape of AAV treatment, emphasizing the potential for reducing the reliance on GC and enhancing the quality of life for AAV patients.
